# Optimization of the Deproteinization Process via Response Surface Methodology, Preliminary Characterization, and the Determination of the Antioxidant Activities of Polysaccharides from *Vitis vinifera* L. SuoSuo

**DOI:** 10.3390/molecules29194734

**Published:** 2024-10-07

**Authors:** Xinnian Ma, Yan Wu, Pei Gao, Qingsong Zheng, Yibo Lu, Fang Yuan, Weixin Jing

**Affiliations:** 1 Department of Biology, School of Basic Medical Sciences, Xinjiang Medical University, Urumqi 830017, China; xinnianma@outlook.com (X.M.); jiyutree@outlook.com (Y.W.); gaopei0202@outlook.com (P.G.); 2 College of Food Science and Nutritional Engineering, China Agricultural University, Beijing 100083, China; qingsong0703@163.com; 3 School of Public Health, Xinjiang Medical University, Urumqi 830017, China; lyb13513837962@outlook.com; 4 Xinjiang Key Laboratory of Molecular Biology for Endemic Diseases, Xinjiang Medical University, Urumqi 830017, China

**Keywords:** *Vitis vinifera* L. SuoSuo, polysaccharides, antioxidant activity, response surface methodology

## Abstract

In this study, the response surface method (RSM) was used to optimize the deproteinization process of polysaccharides from *Vitis vinifera* L. SuoSuo (VTP). The antioxidant capacities of polysaccharides before and after deproteinization were evaluated. The structure of deproteinized VTP (DVTP), which has relatively strong antioxidant activity, was characterized, and the protective effect of DVTP on H_2_O_2_-induced HT22 cell damage was evaluated. The results of the RSM experiment revealed that the ideal parameters for deproteinization included a chloroform/n-butanol ratio (*v*/*v*) of 4.6:1, a polysaccharide/Sevage reagent (*v*/*v*) ratio of 2:1, a shaking time of 25 min, and five rounds of deproteinization. Preliminary characterization revealed that the DVTP was an acidic heteropolysaccharide composed of seven monosaccharides, among which the molar ratio of galacturonic acid was 40.65. FT-IR and the determination of uronic acid content revealed that DVTP contained abundant uronic acid and that the content was greater than that of VTP. In vitro, the antioxidant activity assay revealed that the hydroxyl radical scavenging capacity and total antioxidant capacity of DVTP were greater than those of VTP. In the range of 0.6~0.8 mg/mL, the DPPH scavenging capacities of VTP and DVTP were greater than that of vitamin C. In addition, cell viability was measured via a CCK-8 assay, which revealed that DVTP had a strong defense effect on H_2_O_2_-induced damage to HT22 cells. These findings suggest that DVTP has high antioxidant activity and could be used as a natural antioxidant in functional foods and medicines.

## 1. Introduction

Polysaccharides are macromolecular carbohydrate polymers widely distributed in nature. Polysaccharides isolated from *Dendrobium wardianum* [1], *Ginkgo biloba* [2], *Caulerpa lentillifera* [3], and other plants are receiving increasing amounts of attention because of their antioxidant, antibacterial, antitumor, anti-inflammatory, and other biological activities [4,5]. Most of these products are of the same origin as foods and medicines, have no obvious toxicity or side effects, and have been widely used in various fields, such as biopharmaceuticals, health foods, and cosmetic products. These polysaccharides are expected to be useful for treating diseases as medicinal compounds with great potential for development [6,7,8].

*Vitis vinifera* L. SuoSuo, named after its small berries, is commonly referred to as SuoSuo grape. It belongs to the grape family *V. vinifera* and is a perennial woody (herbaceous) plant.

SuoSuo grapes originated in Corinth, Greece around the 3rd century A.D. The grapes flowed into the Xinjiang region and were cultivated and planted in Turfan, Hotan, Shanshan, and other places. These grapes can be used in medicine to strengthen the spleen and stomach, moisten the lungs, produce saliva, and nourish the blood. Local traditional medicine has been used to treat hepatitis, pediatric measles, and other diseases [9,10]. SuoSuo grapes contain many essential nutrients, such as fructose, glucose, organic acids, vitamins, and amino acids, as well as biologically active substances such as polyphenols, terpenoids, and steroids [11,12]. Research has indicated that polysaccharides from *Vitis vinifera* L. SuoSuo (VTP) exhibit robust neuroprotective properties that inhibit APP mRNA expression by increasing glutathione peroxidase, superoxide dismutase, and catalase activities, thereby decreasing the malondialdehyde content [13,14,15], increasing Bcl-2 mRNA expression, reducing Bax mRNA expression, and inhibiting apoptosis to exert neuroprotective effects [16]. In addition, SuoSuo grape polysaccharides can reduce the level of transforming growth factor-β1 to inhibit CCl_4_-induced hepatic fibrosis in rats [17] and inhibit hepatocellular carcinoma cell proliferation in HepG2 [18]. Additionally, VTP and SuoSuo grape total triterpenes and total flavonoids synergistically antagonize the hepatitis B virus [19].

VTP holds immense promise for development and significant biological roles, offering extensive opportunities in the advancement of therapies and the prevention of diverse ailments. Nevertheless, the majority of research on VTP focuses solely on unrefined polysaccharides, with limited information available regarding the extraction procedure and associated functions. Furthermore, the complete assessment of its medicinal value remains uncertain. To gain a better understanding of the biological activity and mechanism of action of VTP, it is necessary to first purify it. This purification allows for the exploration of its potential value and provides a theoretical foundation for the further development and improved utilization of SuoSuo grapes [12].

Protein is the primary impurity in polysaccharide extract, which has a great influence on the characterization of polysaccharide structure and the determination of biological activity. Therefore, the efficient and rapid removal of proteins from polysaccharides while retaining more undamaged polysaccharide material has become a critical step in polysaccharide purification and has played an important role in subsequent research on polysaccharide bioactivity [20,21]. Therefore, deproteinization is the first crucial step in polysaccharide purification and structure relationship studies. Numerous techniques have been utilized to eliminate protein from polysaccharides, with the Sevage method being the most traditional approach. This method ensures the preservation of the biological structure and activity of polysaccharides [22]. Unfortunately, there are no reports on the optimization of the VTP deproteinization process.

Therefore, in this study, the response surface method (RSM) was used for the first time to study and optimize the deproteinization process of VTP, and the comprehensive score calculated from the protein removal rate and polysaccharide retention rate was used as the evaluation index. A preliminary structural characterization of VTP and deproteinized VTP (DVTP) was then performed via physicochemical properties, Fourier transform infrared spectroscopy (FT-IR), scanning electron microscopy (SEM), and atomic force microscopy (AFM). In addition, vitamin C (Vc) was used as a positive control; the antioxidant properties of VTP and DVTP, including DPPH, hydroxyl radical scavenging ability, and total antioxidant capacity (T-AOC) were investigated. Finally, we evaluated the ability of DVTP to protect against H_2_O_2_-induced oxidative damage to HT22 neurons. Our findings provide a directional basis for subsequent research on the isolation and purification of DVTP and a theoretical reference for the development and utilization of functional foods based on SuoSuo grape polysaccharides.

## 2. Results

### 2.1. Single-Factor Experiments

#### 2.1.1. Effects of the Chloroform/n-Butanol Ratio (*v*/*v*) on Comprehensive Scores of Deproteination

**Figure 1:**
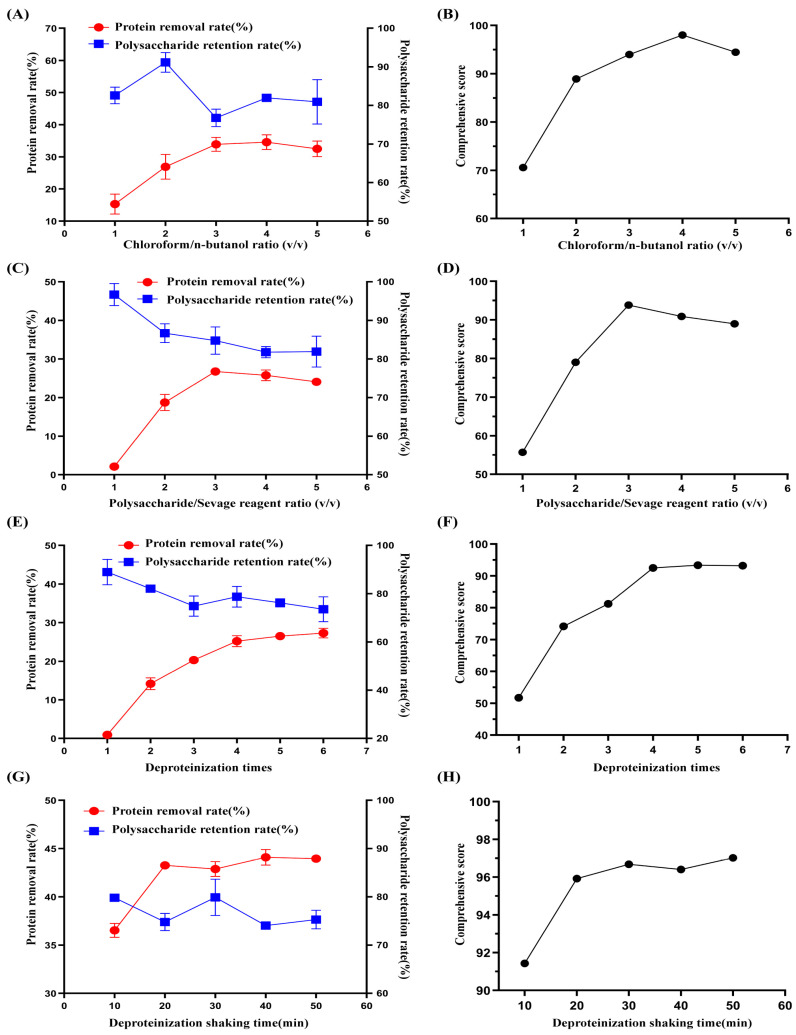
Effects of the chloroform/n-butanol ratio (*v*/*v*). (**A**,**B**) Polysaccharide/Sevage reagent ratio (*v*/*v*), (**C**,**D**) number of deproteinizations, (**E**,**F**) and deproteinization shaking time (**G**,**H**) on the protein removal rate and polysaccharide retention rate.

Figure 1A,B displays the impact of varying chloroform/n-butanol ratios (*v*/*v*) on the rates of polysaccharide preservation and protein removal. With increasing chloroform volume, the retention rate of polysaccharides remained relatively constant, while the rate of protein removal gradually increased, followed by a gradual decrease. The comprehensive deproteinization score reached its highest value at a ratio of 4:1, the polysaccharide retention rate and protein removal rate were 86.23 ± 0.36% and 25.04 ± 1.22%, respectively, and the comprehensive score was 98.03. Hence, for the RSM trial, the central point selected for optimizing the ratio of chloroform/n-butanol (*v*/*v*) was 4:1.

#### 2.1.2. Effect of the Polysaccharide/Sevage Reagent Ratio on the Comprehensive Deproteinization Score

As shown in Figure 1C,D, as the polysaccharide volume increased, the polysaccharide retention rate slowly decreased, the protein removal rate gradually increased and then decreased, and the comprehensive deproteinization score reached its highest value at a ratio of 3:1. The polysaccharide and protein removal rates were 84.79 ± 3.54% and 26.78 ± 0.3%, respectively, with a comprehensive score of 93.84. For the RSM experiment, 3:1 was chosen as the central point for optimizing the polysaccharide-to-Sevage volume ratio.

#### 2.1.3. Effect of the Number of Deproteinizations on the Comprehensive Deproteinization Score

With an increasing number of deproteinizations, the protein removal rate continued to increase slowly, but the polysaccharide retention rate gradually decreased. The comprehensive scores reached their highest values at the fourth deproteinization time, when the polysaccharide retention rate and protein removal rate were 78.72 ± 4.31% and 25.22 ± 1.43%, respectively, and the comprehensive score was 92.50 (Figure 1E,F). Hence, the number of deproteinizations was optimized by selecting four instances of deproteinization as the focal point.

#### 2.1.4. Impact of Shaking Time on the Comprehensive Deproteinization Score

The influence of shaking time on polysaccharide retention and protein removal rate is shown in Figure 1G,H. The retention rate of polysaccharides initially decreased but then increased, whereas the removal rate of proteins increased continuously as the shaking time increased. After 20 min, the comprehensive deproteinization score significantly increased and then reached a plateau. The polysaccharide retention rate and protein removal rates were 74.78 ± 1.78% and 43.26 ± 0.28%, respectively, and the comprehensive score was 95.92. Therefore, 20 min was chosen as the central point for optimizing the shaking time.

### 2.2. Response Surface Analysis

Regression analysis of the Box–Behnken central combinatorial experimental design (BBD) data was performed using Design Expert 13.0.1.0 x64 software, with the comprehensive deproteinization score serving as the response value (Y). The regression model equation was as follows (1):

Y = 89.95 + 2.33A − 2.95B + 2.64C + 3.03D − 0.9643AB + 0.3772AC − 1.05AD − 0.3178BC + 0.6505BD − 0.1798CD − 2.74A^2^ − 1.01B^2^ − 1.72C^2^ − 1.48D^2^ (1)

The comprehensive scores of deproteinization are represented by Y, whereas A, B, C, and D denote four individual factors. Further examination was conducted on the importance of the regression model, Table 1 shows the experimental design of Box-Behnken Center combination and the results of each group, analysis of variance are displayed in Table 2. The significance of the model coefficients was measured via the *F* test and *p* value, with *p* < 0.05 indicating that the model term was significant. The model had a highly significant *F* value of 11.75, with a *p* value less than 0.0001. The lack of fit had an *F* value of 0.8231 and a *p* value of 0.663, indicating that the disparity was not statistically significant (*p* > 0.05) and that the model exhibited strong predictive precision.

According to the regression analysis, the predicted and measured values were strongly correlated, with R^2^ = 0.9320 and R^2^_Adj_ = 0.8527 indicating differences of less than 0.2. Furthermore, the relatively low coefficient of variation (CV) (1.84%) suggested that the measured data were highly precise and dependable. Hence, the regression equation can be further used for the optimization of favorable extraction process conditions, so the model can be used to analyze the variation in response values. Furthermore, as illustrated in Table 2, factors A, B, C, and D, along with factor A^2^, exhibited remarkably significant influences on the composite scores of deproteinization (*p* < 0.01). Additionally, factor C^2^ had a significant effect (*p* < 0.05). The interaction between AB, AC, AD, BC, BD, and CD was not significant (*p* > 0.05).

**Figure 2:**
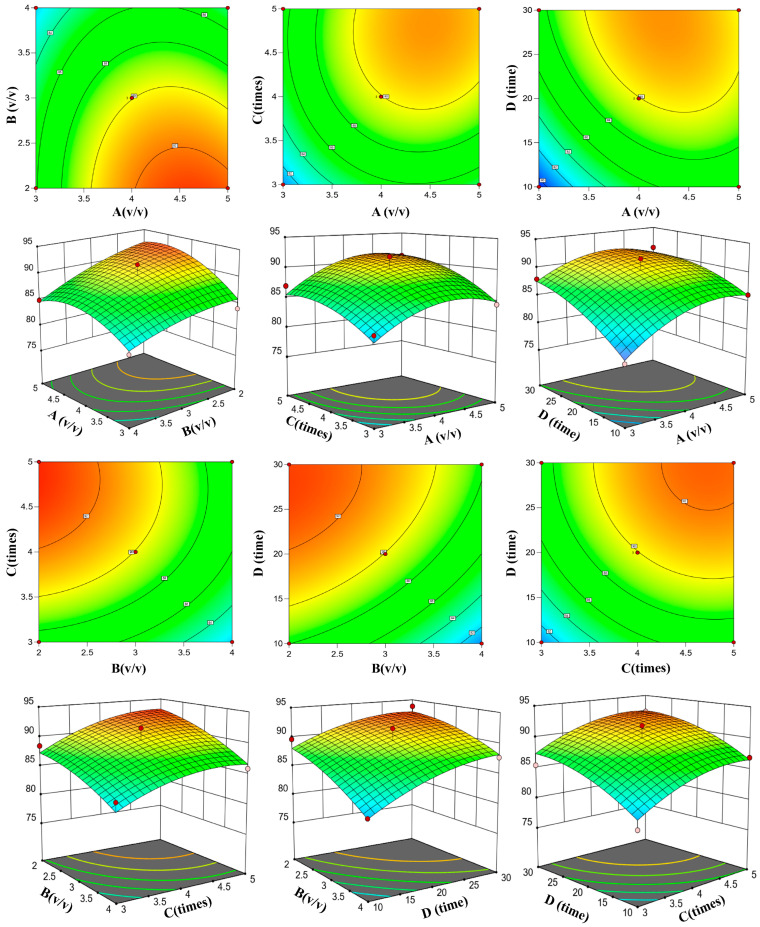
Response surface methodology contour maps and 3D surface plots displaying the effects of variables on the comprehensive deproteinization score. Note: chloroform/n-butanol ratio (*v*/*v*) (A), polysaccharide/Sevage reagent ratio (*v*/*v*) (B), deproteinization times number of deproteinizations (C), and shaking time for deproteinization (D).

Design Expert was used to create the 3D response surfaces and contour plots in Figure 2 to visualize the effect of the two operational parameters on the comprehensive deproteinization scores, with steep surfaces indicating the significance of the impact between variables. The response surfaces depicted in Figure 2 are nearly flat, suggesting that the interaction between the four factors was insignificant. These findings are in line with the regression model analysis, which was also used to identify the optimal conditions for optimization [23]. The following optimal deproteinization conditions were obtained according to the analysis of the response surface: the chloroform/n-butanol ratio (*v*/*v*) was 4.555:1, the polysaccharide/Sevage reagent ratio was 2:1, the shaking time was 25.524 min, and the number of deproteinizations was 4.893. Under these conditions, the comprehensive score for deproteinization was 94.78. We modified the optimal process conditions on the basis of actual deproteinization operations to a chloroform/n-butanol ratio (*v*/*v*) of 4.6:1, a polysaccharide/Sevage reagent ratio (*v*/*v*) of 2:1, a shaking time of 25 min, and five rounds of deproteinization.

To verify that there was no deviation between the predicted results and the measured values, the experimental verification was repeated three times via the modified optimal process conditions. The rate of protein removal was 49.5 ± 1.3%, while the rate of polysaccharide retention was 75.57 ± 1.35%. Deproteinization achieved a comprehensive score of 97.86, with an error of only 3.08%, whereas the predicted value was 94.78. By further analyzing the 3D response surfaces and 2D contours and combining the ANOVA results, the effects of the four factors on the comprehensive scores of deproteinization can be ranked as follows: deproteinization shaking time (D) > polysaccharide/Sevage reagent ratio (*v*/*v*) (B) > deproteinization time (C) > chloroform/n-butanol ratio (*v*/*v*) (A).

### 2.3. Analysis of Physicochemical Properties and Structural Characterization of Polysaccharides

#### 2.3.1. Content of Uronic Acid in Polysaccharides

**Figure 3:**
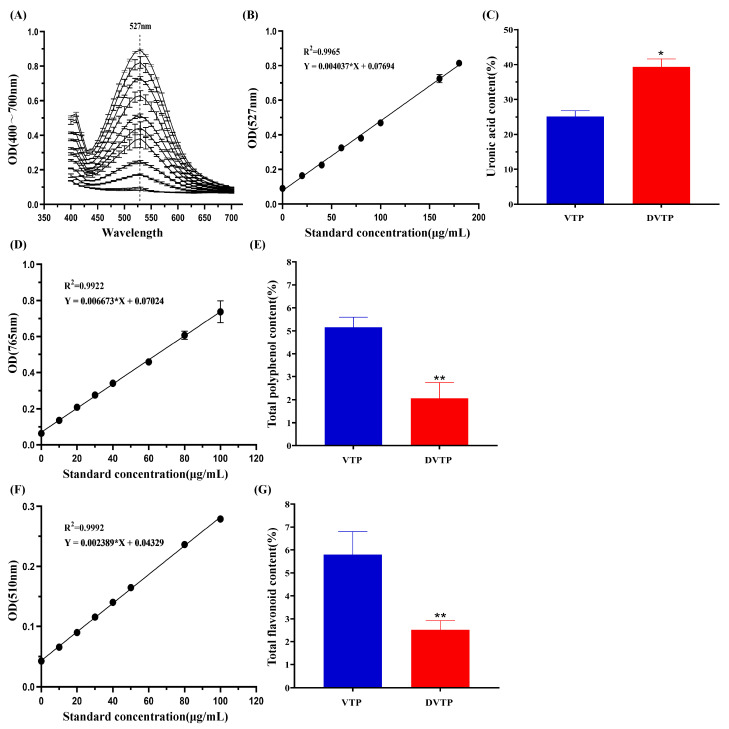
(**A**) Full wavelength scanning of glucuronic acid standard products; (**B**) standard curve of glucuronic acid standard products; (**C**) statistical diagram of uronic acid content in VTP and DVTP; (**D**) standard curve of gallic acid standard products; (**E**) statistical diagram of total polyphenol content in VTP and DVTP; (**F**) standard curve of rutin standard products; (**G**) statistical diagram of total flavonoid content in VTP and DVTP; * *p* < 0.05, ** *p* < 0.01 vs. VTP.

As shown in Figure 3A, based on the full wavelength scanning at 400~700 nm, the absorbance value of the glucuronic acid standard product was determined to be the highest at 527 nm. Therefore, 527 nm was taken as the OD value measured in the experiment, and the standard curve was obtained according to the concentration of the glucuronic acid standard product and its corresponding absorbance value: Y = 0.004037, X = 0.07694, and R^2^ = 0.9965. The absorbances of the VTP and DVTP samples were substituted into the standard curve, and the contents of uronic acid in the VTP and DVTP samples were 25.14 ± 1.63% and 39.34 ± 2.29%, respectively.

#### 2.3.2. Determination of Total Polyphenol Content (TPC)

The standard curve was obtained by the concentration of the gallic acid standard product and its corresponding absorbance value (Figure 3D): Y = 0.006673X + 0.07024, R^2^ = 0.9922. The absorbance of the VTP and DVTP samples was substituted into the standard curve, and the content of TPC in VTP and DVTP was 5.16 ± 0.43% and 2.06 ± 0.69%, respectively (Figure 3E).

#### 2.3.3. Determination of Total Flavonoid Content (TFC)

The standard curve was obtained by the concentration of the rutin standard product and its corresponding absorbance value (Figure 3F): Y = 0.002389X + 0.04329, R^2^ = 0.9992. The absorbance of the VTP and DVTP samples was substituted into the standard curve, and the content of TPC in VTP and DVTP was 5.79 ± 1.01% and 2.52 ± 0.41%, respectively (Figure 3G).

#### 2.3.4. FT-IR Spectra Analysis

**Figure 4:**
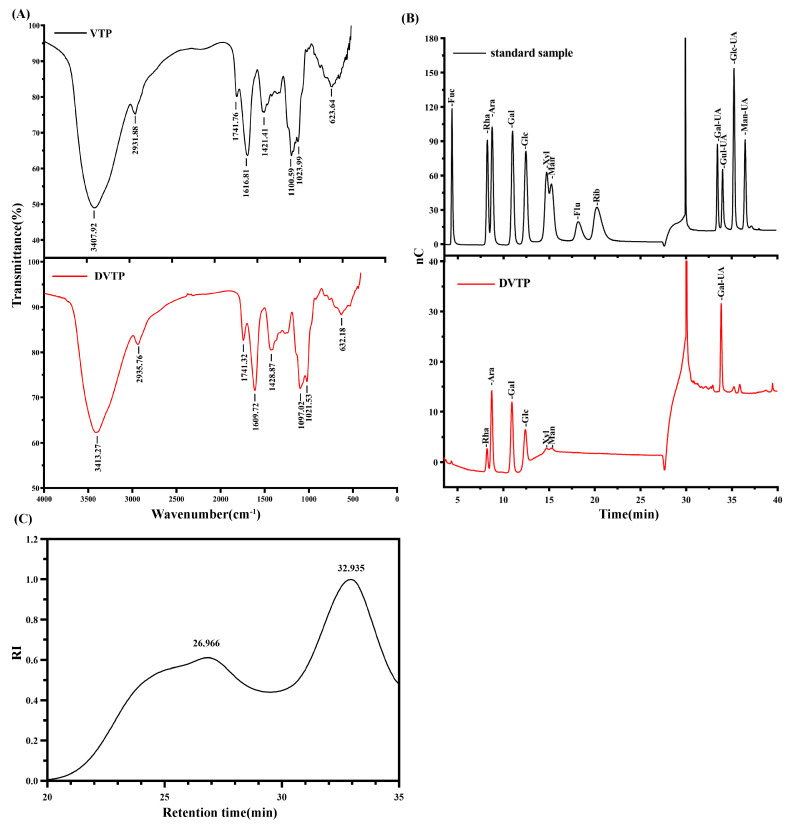
(**A**) Infrared spectra of VTP and DVTP; (**B**) monosaccharide standard and DVTP ion chromatograms; (**C**) HPGPC chromatogram of DVTP.

According to the results of the infrared spectrometer analysis, as shown in Figure 4A, VTP and DVTP have typical polysaccharide characteristic peaks, with strong absorption peaks at 3407.92 cm^−1^ and 3413.21 cm^−1^, which are attributed mainly to O–H stretching vibrations, and the absorption peaks at 2931.88 cm^−1^ and 2935.76 cm^−1^ represent the stretching of C–H bonds. The bands at 1741.76 cm^−1^, 1741.32 cm^−1^, 1616.81 cm^−1^, and 1609.72 cm^−1^ were attributed to the stretching vibrations of C=O and COO-, respectively, indicating that the polysaccharides contained abundant uronic acid, which was consistent with the results of the monosaccharide composition analysis. The peaks at 1421.41 cm^−1^ and 1428.87 cm^−1^ represent the C–H deformation vibration. In addition, the absorption bands at 1100.59 cm^−1^, 1097.02 cm^−1^, 1023.99 cm^−1^, and 1021.53 cm^−1^ indicate that the polysaccharide was in the form of pyranose. The results of the infrared spectrum of polysaccharide show that VTP and DVTP are typical polysaccharides with typical absorption peaks.

#### 2.3.5. Compositional Analysis and Molecular Weight Determination of DVTP Monosaccharides

The molecular weight distribution of the polysaccharides in DVTP consisted of two components (Figure 4C). The standard curve equation established from the Pullulan standard sample was Y = −1.5425X + 15.982 (R^2^ = 99.54). The average molecular weights of DVTP were 160.967 kDa and 1.647 kDa, and the retention times were 26.966 and 32.935 min, respectively. Furthermore, ion chromatography demonstrated that DVTP comprises Rha, Ara, Gal, Glc, Xyl, Man, and Gal-UA in molar proportions of 6.12, 19.31, 16.76, 11.82, 2.42, 2.92, and 40.65, respectively (Figure 4B).

#### 2.3.6. SEM and AFM Analyses

**Figure 5:**
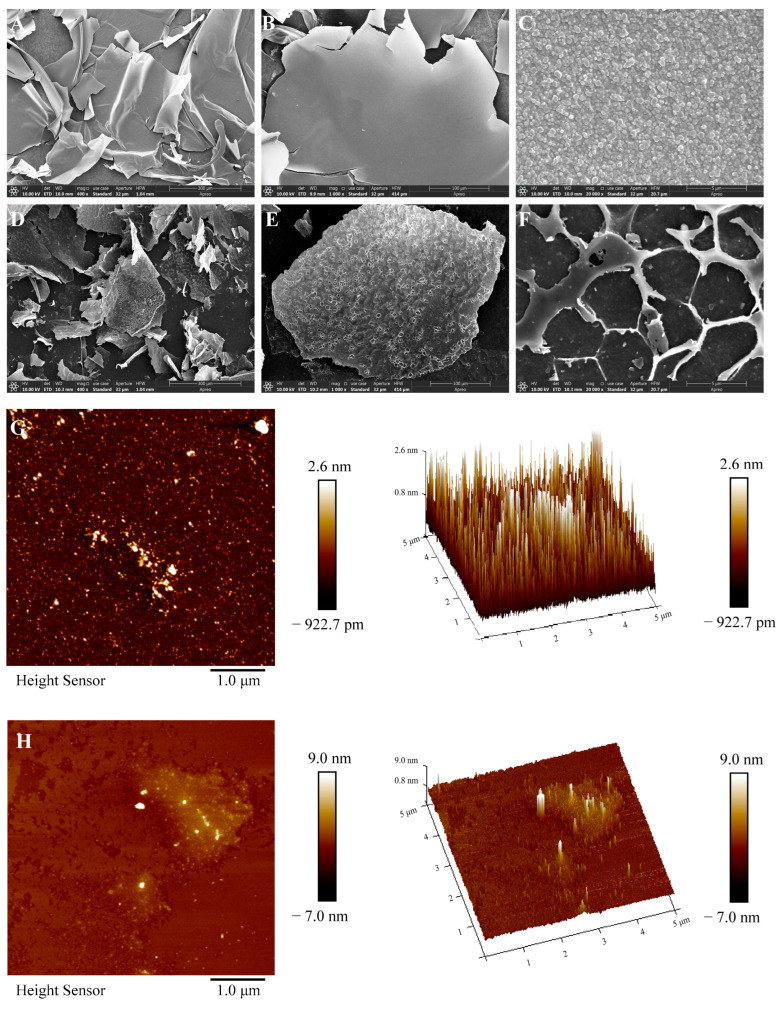
Scanning electron micrographs of VTP (**A**–**C**) and DVTP (**D**–**F**); atomic force microscopy images of VTP (**G**) and DVTP (**H**).

SEM is considered an effective and direct technique for observing the surface of objects [24]. The morphology of VTP and DVTP was studied via SEM. As shown in Figure 5A–C, the surface of VTP presents an irregular sheet-like structure with a rough surface and many circular flakes attached. DVTP also has an irregular sheet-like structure. Notably, the surface of DVTPs present a honeycomb structure with many obvious voids (Figure 5D–F). A larger space in the thin layer can provide a cohesive space for water molecules, increasing their solubility and thus improving their activity [25].

AFM is a high-resolution tool that is widely used in the characterization of polysaccharide surface morphology [26]. As shown in Figure 5F,G, the AFM images of VTP and DVTP show uneven granular and blocky structures. In three-dimensional images, both VTP and DVTP are flame-like aggregates with multiple chains arranged closely, but there are significantly more VTP aggregates than DVTP aggregates. SEM and AFM analyses revealed that the ultrastructure’s of VTP and DVTP were significantly different, which may be due to changes in the microscopic morphology of polysaccharides caused by deproteinization.

### 2.4. In Vitro Antioxidant Activities of the Polysaccharides

**Figure 6:**
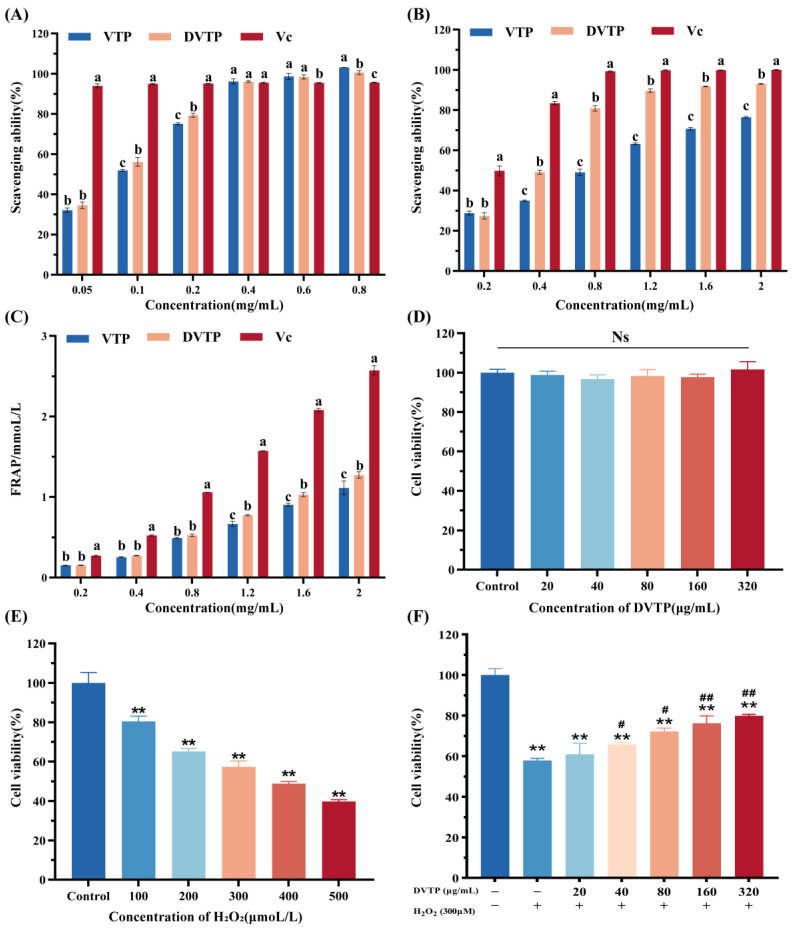
DPPH radical scavenging activity (**A**); hydroxyl radical scavenging activity (**B**); total antioxidant capacity (**C**); ^a–c^ the values with different superscripts are significantly different (*p* < 0.05); effects of DVTP on HT22 cell viability (**D**); effects of H_2_O_2_ on HT22 cell viability (**E**); effects of DVTP on H_2_O_2_-induced injury-related HT22 cell viability (**F**). ** *p* < 0.01 compared with the control groups; # *p* < 0.05, ## *p* < 0.01 compared with the H_2_O_2_-treated group.

As shown in Figure 6A–C. In this study, the capacity to scavenge DPPH and hydroxyl radicals, as well as the T-AOC, tended to increase as the concentrations of both polysaccharides increased. The ability of VTP and DVTP to scavenge T-AOC and hydroxyl free radicals was lower than that of Vc at all the tested concentrations, but the ability of VTP and DVTP to scavenge DPPH free radicals was greater than that of Vc in the concentration range of 0.6 to −0.8 mg/mL (*p* < 0.05). At a concentration of 0.8 mg/mL, the DPPH scavenging capacity of VTP, DVTP, and Vc reached maximum values of 103.13 ± 0.07%, 100.57 ± 1%, and 95.66 ± 0.14%, respectively (Figure 6A).

Notably, in the range of 0.4~2.0 mg/mL, the ability of DVTP to scavenge hydroxyl free radicals was significantly greater than that of VTP (*p* < 0.05) (Figure 6B). At a concentration of 2.0 mg/mL, the maximum hydroxyl radical scavenging capacities of VTP, DVTP, and Vc were 76.41 ± 0.46%, 93.05 ± 0.16%, and 100.02 ± 0.09%, respectively. Similarly, the T-AOC of DVTP slightly exceeded that of VTP at a concentration of 1.2~2.0 mg/mL; at a concentration of 2.0 mg/mL, the T-AOC values for VTP, DVTP, and Vc were 1.11 ± 0.09 mM, 1.27 ± 0.04 mM, and 2.57 ± 0.06 mM, respectively (Figure 6C).

### 2.5. Cell Viability Assay

After incubation with various concentrations of DVTP for 24 h, there was no significant difference in cell viability compared with the normal control group at concentrations ranging from 20 to 320 μg/mL (*p* > 0.05), indicating that DVTP did not exhibit cytotoxicity to HT22 cells. The subsequent induction of oxidative damage by H_2_O_2_ resulted in a gradual decrease in cell viability, with the survival rate reaching approximately 57.34 ± 2.97% at a H_2_O_2_ concentration of 300 μM, which was used for further experiments. Following pretreatment with DVTP for 4 h under oxidative damage induced by H_2_O_2_, the survival rate of HT22 cells increased significantly to 60.91 ± 5.51%, 65.83 ± 0.85%, 72.24 ± 1.6%, 76.27 ± 3.67%, and 79.87 ± 0.79%, respectively. These results demonstrate that DVTP effectively mitigated the cytotoxic effects induced by H_2_O_2_, protected cells from oxidative damage, and promoted cell regrowth or proliferation, as shown in Figure 6F.

## 3. Discussion

The deproteinization process of VTP was successfully optimized via RSM in this study. The optimal extraction conditions were as follows: a ratio of 4.6:1 for chloroform/n-butanol (*v*/*v*), a ratio of 2:1 for polysaccharide/Sevage reagent (*v*/*v*), and a shaking time of 25 min; deproteinization was performed a total of five times. The actual comprehensive deproteinization score was 97.86, and the error in the prediction result was only 3.08%, which indicated that the prediction effect was good. Among the four experimental factors, the comprehensive score of deproteinization was most affected by shaking time, which aligns with the results of previous studies on the deproteinization of *Solanum tuberoides* polysaccharides [27] and *Bletilla striata* polysaccharides [28], where shaking time was identified as the primary factor. These findings suggest that attention should be given to the duration of shaking during the deproteinization of polysaccharides.

Upon calculating the molecular mass of DVTP, it was discovered that the distribution of the molecular weights of the polysaccharides of DVTP comprised two components. This means that DVTP is not a uniform polysaccharide, and the subsequent isolation and purification of the polysaccharide after the removal of the protein is necessary. The monosaccharide composition and the content of uronic acid were determined, which indicated that DVTP is an acidic heteropolysaccharide that is abundant in Gal-UA, and the content of uronic acid in DVTP was significantly greater than that in VTP (*p* < 0.05). There are three possible reasons for this phenomenon: 1. During polysaccharide extraction, the presence of proteins may interfere with the results of the uronic acid detection. Therefore, after the removal of the protein, the content of uronic acid, which was originally masked or interfered with by the protein, can be more accurately detected, thus showing an increase in the content of uronic acid [29]. 2. Some uronic acids may be present in bound forms in polysaccharide–protein complexes [30]. When the protein is removed, these bound forms of uronic acid are released as a free state, resulting in increased levels of uronic acid detected. 3. After polysaccharides were deproteinized, the proportion of protein content decreased, the proportion of total sugar content increased, and the corresponding uronic acid increased. After further measurement of TPC and TFC, it was found that the TPC and TFC of DVTP were significantly lower than that of VTP (*p* < 0.01); these findings confirm that deproteinization plays an important role in the separation and purification of polysaccharides.

According to previous research reports, the examination of polysaccharide fractions derived from *Cistanche deserticola* indicated that the amount of Gal-UA in CDP-C was greater than that in CDP-B, and that in CDP-A and CDP-C presented the greatest antioxidant activity in vitro. Furthermore, this biological function could be attributed to the elevated Gal-UA content in CDP-C, as stated in [31]. The fraction with a relatively high uronic acid content among *Ziziphus jujuba* polysaccharides presented relatively high antioxidant activity [32]. The polysaccharide components of ZSP3c and ZSP4b were isolated and purified from *Z. jujuba cv*. Jinsixiaozao, which has stronger free radical scavenging activity than ZSP1b without uronic acid in the polysaccharide fraction [33]. Among the polysaccharide components of *Camellia fascicularis* leaves, PCFc-1 contained more Gal-UA than PCFa-1 and had a greater antioxidant capacity [23].

The above results suggest that polysaccharides with relatively high uronic acid contents have a greater antioxidant capacity than polysaccharides with lower uronic acid contents, and uronic acid may be an effective indicator of the antioxidant capacity of polysaccharides [34]. Therefore, DVTP may have a relatively high antioxidant capacity. Nevertheless, the antioxidant effectiveness of polysaccharides is commonly impacted by a blend of elements, necessitating additional research to elucidate the correlation between their structural configuration and antioxidant efficacy [35]. To verify whether the optimized VTP deproteinization process will cause a loss of biological activity and whether DVTP with higher uronic acid content has a stronger antioxidant capacity, this study evaluated the antioxidant properties of VTP and DVTP through in vitro experiments.

The DPPH radical stands out as one of the few free radicals that is highly stable; it has been widely used to evaluate the oxygen radical scavenging activity of natural antioxidants such as polysaccharides, flavonoids, and polyphenols [34]. Polysaccharides may reduce DPPH free radicals through the hydrogen supply of the hydroxyl group to scavenge DPPH [36]. In this study, starting at 0.6 mg/mL, the capacity of VTP and DVTP to scavenge DPPH free radicals was slightly greater than that of Vc. This difference was notably greater than the scavenging ability of SuoSuo grape crude polysaccharides reported previously [37]. Compared with other types of natural polysaccharides, VTP and DVTP also had greater scavenging capacities for DPPH free radicals than the dandelion root homopolysaccharides DRP-3a (1.0 mg/mL, 71.58 ± 3.11%) [38], *cordyceps cicadae* polysaccharides CP70 (0.8 mg/mL, 81.67%) [39], and *Siraitia grosvenorii* homopolysaccharides SGP (2 mg/mL, 89.46 ± 0.14%) [40]. These findings suggest that VTP and DVTP possess highly potent DPPH scavenging capabilities and that the deproteinization procedure did not impact their ability to neutralize DPPH radicals.

Hydroxyl radicals, which are the most reactive free radicals, can effortlessly traverse cell membranes and readily interact with nearly all biomolecules (such as carbohydrates, lipids, proteins, and DNA) present in cells. This interaction ultimately results in the onset of aging, cancer, and numerous other diseases. Hence, the elimination of hydroxyl radicals is crucial for safeguarding biological systems [41]. Polysaccharides may play a role in this process by utilizing the hydroxyl group in the reducing agent to offer electrons for stabilizing free radicals and by binding with them to halt the chain reaction of free radicals [42,43]. The ability of both VTP and DVTP to scavenge hydroxyl radicals increased as the concentration increased in the range of 0.2~2.0 mg/mL, and both were less effective than Vc in scavenging. Interestingly, the hydroxyl radical scavenging ability of DVTP was significantly greater than that of VTP in the concentration range of 0.4~2.0 mg/mL. The maximum scavenging capacities of VTP, DVTP, and Vc at 2.0 mg/mL were 76.41 ± 0.46%, 93.05 ± 0.16%, and 100.02 ± 0.09%, respectively. The percentage of hydroxyl radical scavenged by the 5.5 mg/mL crude polysaccharide *Gentiana scabra bge* was reported to be 29.53% [44], the percentage of hydroxyl radical scavenged by 5.0 mg/mL *Sargassum pallidum* homopolysaccharides PSP-1 was reported to be 64.84% [45], and the percentage of hydroxyl radical scavenged by 2 mg/mL *Porphyra haitanensis* homopolysaccharides PHP3 was 22.84% [46]. Thus, the hydroxyl radical scavenging ability of VTP and DVTP is greater than that of other polysaccharides previously reported. In addition, after deproteinization, DVTP has a relatively strong hydroxyl radical scavenging ability. DVTP can be considered a good hydroxyl radical scavenging agent and has a protective effect on oxidative damage to a certain extent.

In routine analyses of plant extracts, ferric-reducing antioxidant power (FRAP) is often referred to as the “total antioxidant capacity” [47]. At all the experimental concentrations, the T-AOC of VTP and DVTP was lower than that of Vc in the concentration range of 0.8~2.0 mg/mL. The total antioxidant capacities of VTP and DVTP at 1.2 mg/mL were 0.67 ± 0.03 mM and 0.77 ± 0.01 mM, respectively, which are lower than those of *Auriculariales* polysaccharides, whose T-AOC was 1.1 mM at the same concentration [48]. The results indicate that there is a need for improvement in the T-AOC of both VTP and DVTP. Notably, at a concentration of 1.2~2.0 mg/mL, the T-AOC of DVTP was slightly greater than that of VTP.

Research has indicated that when deproteinizing, it is important to consider not only the efficiency of deproteinization and the rate of polysaccharide loss but also the impact on antioxidant activity [21]. During the process of deproteinization, certain elements that possess antioxidant properties or elements that increase the antioxidant activity of polysaccharides are eliminated, consequently diminishing the antioxidant activity of the polysaccharide solution. As a result, nondeproteinized polysaccharides exhibit greater antioxidant activity than deproteinized polysaccharides. According to previous reports, the antioxidant activity of nondeproteinized *Tremella* polysaccharides and *Lentinus edodes* stipe polysaccharides is greater than that of deproteinized *Tremella* polysaccharides and *Lentinus edodes* stipe polysaccharides [49,50]. The evaluation of this study involved both the rate of protein removal and the rate of polysaccharide retention as indices. These indices aimed to maximize the retention of polysaccharide yield and bioactivity while also ensuring the protein removal rate [21,22]. In this study, DVTP had a greater hydroxyl radical scavenging capacity and total antioxidant capacity than VTP, and the DPPH free radical scavenging capacity of DVTP was superior to that of VTP in the range of 0.05~0.2 mg/mL. These results indicate that although both VTP and DVTP have good antioxidant activity, after deproteinization, DVTP has stronger antioxidant activity, which further indicates that the optimized Sevage deproteinization process does not destroy the antioxidant activity of polysaccharides, and it increases the biological activity of polysaccharides, indicating the importance and significance of this work.

Oxidative stress-induced nerve damage and cell death are two of the characteristics and main mechanisms of neurodegenerative pathologies [51]. H_2_O_2_ is a naturally occurring oxidative metabolic byproduct in biological systems. Exogenous H_2_O_2_ readily diffuses into cells after entry due to its high membrane permeability, causing damage to cellular structures, and oxidative stress can be induced in neurons by H_2_O_2_ treatment [52,53,54]. In H_2_O_2_-induced oxidative damage, the viability of HT22 cells increased with increasing polysaccharide concentration. DVTP may protect HT22 cells from H_2_O_2_-induced oxidative damage through its antioxidant activity. Previous studies have shown that Asteris Radix et Rhizoma polysaccharide-1 protects PC12 neurons from H_2_O_2_-induced oxidative stress [55]. Polysaccharides from *Millettia dielsiana* MDP1 and S-MDP1 had protective effects on H_2_O_2_-induced PC12 cell death [56]. The polysaccharide *Pleurotus sajor-caju*, PSP2-1, significantly improved the viability of H_2_O_2_-induced oxidatively damaged HT22 neurons [57]. The above studies indicate that polysaccharides effectively antagonize the oxidative damage of neurons, but the exact molecular mechanism is still unclear, and the specific mechanism needs to be further studied.

The findings from these experiments indicate that the Gal-UA-rich acid heteropolysaccharides found in SuoSuo grapes have the potential to be utilized as natural antioxidants. Furthermore, the bioactivity of these polysaccharides could be improved through the ongoing optimization of the production process. By conducting a comprehensive analysis of the structure and biological role of polysaccharides, the physicochemical properties, structural characterization, and potential mechanism of action of SuoSuo grape polysaccharides should be further clarified to facilitate their complete utilization and advancement, which would promote the subsequent extraction and purification of SuoSuo grapes and the investigation of their biological properties.

## 4. Materials and Methods

### 4.1. Materials and Reagents

SuoSuo grapes were purchased from the Turpan Uyghur Medicinal Materials Market in the Xinjiang Uygur Autonomous Region. HT22 cells were acquired from PLST Co., Ltd. (Wuhan, China). The NJJC Bioengineering Institute (Nanjing, China) provided a DPPH kit (A153-1), a hydroxyl radical kit (A018-1-1), and a T-AOC kit (A015-3-1). CCK-8 (BA00208) was obtained from Beijing Biosynthesis Biotechnology Co., Ltd. (Beijing, China). Beijing Solebao Technology Co., Ltd. (Beijing, China) supplied a dialysis bag (YA1078). Anhydrous ethanol concentrated sulfuric acid, phenol, bovine serum albumin, glucose, and other reagents were all of analytical or chemical purity.

### 4.2. Sample Preparation

The SuoSuo grapes were washed, dried at 105 °C, pulverized, and sieved through an 80-mesh sieve. The powder was grouped and packaged (5 g/packet). The mixture was then placed inside a Soxhlet extractor and subjected to reflux with petroleum ether, ethyl acetate, and anhydrous ethanol for 10 h to eliminate impurities that are soluble in fats and alcohols [58]. The processed SuoSuo grape powder was evaporated to dryness in a ventilated area.

The SuoSuo grape powder was extracted by hot water extraction, and the filtered liquid was then collected and centrifuged at a speed of 4000 revolutions per minute for 10 min. The resulting liquid was collected and concentrated. Next, anhydrous ethanol was introduced to modify the alcohol volume fraction of the solution to 75%, followed by an overnight settling at a temperature of 4 °C. Following the process of alcohol precipitation, the resulting solid was gathered, subjected to centrifugation, dissolved in a suitable quantity of water, enclosed within dialysis pouches, subjected to dialysis via flow of tap water for 24 h, and followed by distilled water for another 24 h to eliminate tiny particles and inorganic ions [59]. The dialyzed solution was concentrated under reduced pressure and freeze-dried under vacuum to obtain VTP.

### 4.3. Analytical Methods

The VTP deproteinization process was optimized in terms of the polysaccharide retention rate and protein removal rate. The phenol–sulfuric acid method was used to determine the overall polysaccharide amount, with glucose utilized as the reference standard [60]. A standard of bovine serum albumin was used to determine the protein content according to the Bradford method [20]. By applying Formula (2) [61], comprehensive scores of VTP deproteinization were obtained after the polysaccharide retention rate (X) and protein removal rate (Y) were analyzed.

Comprehensive score = (X/X_max_ + Y/Y_max_) ⨉ 0.5 (2)

### 4.4. Experimental Design

#### 4.4.1. Single-Factor Experimental Design

To optimize the deproteinization of SuoSuo grape polysaccharides, the chloroform/n-butanol ratio (*v*/*v*) (A), polysaccharide/Sevage reagent ratio (*v*/*v*) (B), number of deproteinizations (C), and shaking time for deproteinization (D) were selected. A single-factor design was adopted: The ratios of chloroform/n-butanol (*v*/*v*) used were 1:1, 2:1, 3:1, 4:1, and 5:1. The ratios of polysaccharide to Sevage reagent (*v*/*v*) used were 1:1, 2:1, 3:1, 4:1, and 5:1. The deproteinization time was 5 min, and the shaking time ranged from 10 to 50 min. During the optimization procedure for the experimental variables, while altering one variable in every trial, the remaining variables were kept constant: the chloroform/n-butanol ratio (*v*/*v*) was 4:1, the polysaccharide/Sevage reagent ratio (*v*/*v*) was 3:1, the number of deproteinizations was set at 4, and the shaking time was 10 min.

#### 4.4.2. Experimental Design through RSM

The influence of four individual factors on the comprehensive scores of VTP deproteinization was investigated via the BBD-RSM method. This investigation was based on a single-factor experiment, and the experimental design can be found in Table 3.

### 4.5. Structural Characterization of Polysaccharides

#### 4.5.1. Determination of Uronic Acid Content

First, a series of standard solutions, carbazole solutions, and sodium tetraborate concentrated sulfuric acid solutions were prepared, and finally, 0.1 mg/mL VTP and DVTP solutions were prepared. Next, 5 mL of sodium tetraborate-concentrated sulfuric acid reagent was added dropwise to a 1 mL standard solution and polysaccharide sample in an ice bath and then shaken to ensure that the mixture was completely blended. The test tubes were placed in boiling water for 20 min to allow chemical reactions to take place and then cooled to room temperature upon completion. Then, a 0.15% carbazole solution (0.2 mL) was poured into each test tube, fully mixed by shocking, and left to sit for 60 min. Then, the standard solution was scanned at a full wavelength of 400~700 nm with an interval of 1 nm via a microplate reader, suitable values were selected as the subsequent detection wavelength, and finally, the concentration of glucuronic acid was taken as the horizontal coordinate. The absorbance values were plotted as standard curves, and the content of glucuronic acid in VTP and DVTP was calculated according to the standard curves [62].

#### 4.5.2. Determination of TPC

Take 1.0 mL polysaccharide sample (mass concentration: 100 µg/mL) and gallic acid standard solution (mass concentration: 0, 10, 20, 30, 40, 50, 60, 80, 100 µg/mL), add Folin reagent, 5.0 mL in each, and shake well for 5 min. Add 4.0 mL of 7.5% sodium carbonate solution. After 60 min of light avoidance, measure absorbance at 765 nm [63].

#### 4.5.3. Determination of TFC

Rutin standard solution with 50 μL mass concentration of 0, 10, 20, 30, 40, 50, 60, 80, 100 mg/mL and a polysaccharide sample with 100 µg/mL mass concentration (prepared with 30% ethanol solution by volume fraction) were taken, and a 15 μL of NaNO_2_ solution with 5% mass fraction was added. These were shaken well and allowed to stand for 6 min; AI(NO_3_)_3_ solution with 5% mass fraction of 30 μL was added and allowed to stand for 6 min; 105 μL of NaOH solution with 4% mass fraction was added and left for 15 min; and absorbance was measured at 510 nm [64].

#### 4.5.4. Monosaccharide Composition Determination

Five milligrams of the DVTP sample were accurately weighed in a chromatographic vial, and 1 mL of a 2 M trifluoroacetic acid (TFA) solution was added. Ultrapure nitrogen gas was used to evaporate the TFA after the mixture was heated at 121 °C for 2 h. Then, 99.99% methanol was added for cleaning, and the samples were dried under ultrapure nitrogen. The process of cleaning with methanol was performed two to three times. To measure the dissolved material, sterile water was added and passed through a 0.22 μm pore-size filter.

To analyze and detect the monosaccharide fractions, an electrochemical detector and a Thermo ICS 5000+ ion chromatography system (Thermo Fisher Scientific, Waltham, MA, USA) was employed. The experiment utilized a Dionex™ CarboPac™ PA20 chromatographic column (Thermo Fisher Scientific, Waltham, MA, USA) with dimensions of 150 mm × 3.0 mm and a concentration of 10 μM. The volume of the injection mixture was 5 μL, and the mobile phases consisted of H_2_O (mobile phase A), 0.1 M NaOH (mobile phase B), and 0.1 M NaOH with 0.2 M NaAc (mobile phase C). The flow rate was set at 0.5 mL/min, and the column temperature was maintained at 30 °C. The analysis of the monosaccharide components was carried out with fucose (Fuc), rhamnose (Rha), arabinose (Ara), galactose (Gal), glucose (Glc), xylose (Xyl), mannose (Man), fructose (Fru), ribose (Rib), galacturonic acid (Gal-UA), glucuronic acid (Glc-UA), mannuronic acid (Man-UA), and guluronic acid (Gul-UA), which were used as standards and analyzed under the same experimental conditions. By comparing the retention times with those of the standards, the constituent monosaccharides of DVTP were identified [65].

#### 4.5.5. FT-IR Analysis

One hundred thirty mg of potassium bromide powder was mixed with 1.3 mg of a VTP or DVTP sample for grinding treatment (with a sample-to-potassium bromide mass ratio of 1:100), fully ground in an agate grinding bowl, poured into a mold, pressed into a transparent sheet with a tablet press (pressure of 8 tons), and detected with an FT-IR spectrometer. The wavenumber range was 400~4000 cm^−1^, the spectrometer resolution was 4 cm^−1^, and the signal-to-noise ratio was 50,000:1. The samples were scanned 32 times to obtain the infrared absorption spectra of VTP and DVTP [66].

#### 4.5.6. Molecular Weight Determination

The molecular weights of the polysaccharides were determined via high-performance gel permeation chromatography (HPGPC) [67]. The chromatographic system used was a high-performance liquid chromatography system (U3000, Thermo Fisher Scientific, USA) equipped with a differential refractive index detector (Optilab T-rEX, Wyatt Technology Co., Goleta, CA, USA) and a gel exclusion column (300 mm × 8 mm, Shodex OH-pak SB-805 and 803).

The sample was dissolved in a 0.1 M NaNO_3_ aqueous solution (containing 0.02% NaN_3_, *w*/*w*) at a final concentration of 1 mg/mL and passed through a 0.45 μm pore-size filter membrane. The 100 μL sample was subsequently detected, and elution was performed for 75 min at a column temperature of 45 °C and a flow rate of 0.6 mL/min. The molecular weight of DVTP was calculated via calibration curve equations for Pullulan polysaccharide standards with different molecular weights (3.65, 21, 131.4, 610.5, 821.7, and 3755 kDa) of the same concentration as the sample.

#### 4.5.7. SEM Analysis

Using a vacuum spraying instrument, the sample was placed on the sample table approximately 10~15 cm away from the evaporation source for rotation, gold spraying (10 kV for 60 s), and uniform spraying. After completion, the sample was scanned via SEM (Apreo C, Thermo Fisher Scientific, Waltham, MA, USA), the scanning data of each multiple were recorded, and the image was further analyzed [68].

#### 4.5.8. AFM Analysis

The VTP and DVTP samples were prepared in a 1 μg/mL solution with deionized water and dispersed via 100 W ultrasonication for 3 min. A 5 μL ultrasonically dispersed solution was added dropwise to a clean mica sheet, and the mica sheet was dried under ambient air pressure, with a scanning range of 5 μm × 5 μm. Under a frequency of 1.00 Hz, the sample was placed in the AFM instrument (Dimension Icon, Bruker, Bremen, Germany) for scanning, and the image was further analyzed by NanoScope Analysis 1.8 [69].

### 4.6. Antioxidant Activity of VTP and DVTP In Vitro

#### 4.6.1. DPPH Radical Scavenging Test

The DPPH radical scavenging capacities of VTP and DVTP were determined by a kit, and the operation steps were carried out according to the manufacturer’s instructions [70]. The concentrations of the VTP and DVTP solutions were 0.05, 0.1, 0.2, 0.4, 0.6, and 0.8 mg/mL, and the positive control was a Vc solution within the same concentration range. The scavenging capacity was calculated via the formula provided below (3).

Scavenging activity (%) = [1 − (A_sample_ − A_control_)/A_blank_] × 100 (3)

The absorbances of the sample and DPPH solution are represented by A_sample_, while A_control_ represents the absorbance of the sample solution without DPPH, and A_blank_ represents the absorbance of the DPPH solution.

#### 4.6.2. Hydroxyl Radical Scavenging Test

The abilities of VTP and DVTP to scavenge hydroxyl radicals were assessed via a kit [48] following the steps outlined in the reagent kit’s instructions. Solutions of VTP and DVTP were made with concentrations ranging from 0.20~2.00 mg/mL. A Vc solution within the same concentration range served as the positive control. The scavenging capacity was calculated via the formula provided below (4).

Scavenging activity (%) = (A_control_ − A_sample_)/(A_control_ − A_blank_) × 100 (4)

The absorbance of the sample is represented by A_sample_, whereas A_control_ represents the absorbance of the water instead of the sample. The abbreviation “A_blank_” refers to the absorbance of the reagent blank without sodium salicylate.

#### 4.6.3. T-AOC Test

The T-AOC was determined with a FRAP kit according to the manufacturer’s instructions [48]. A standard curve was prepared using FeSO_4_-7H_2_O as the reference, and the linear regression equation Y = 3.367X + 0.01792 (R^2^ = 0.9997) was derived, where Y represents the concentration of Fe^2+^ and X represents the absorbance of the sample. The concentrations of the SuoSuo grape polysaccharide solutions were 0.20, 0.40, 0.80, 1.20, 1.60, and 2.00 mg/mL. The positive control was a Vc solution in the same concentration range. The concentration of the FeSO_4_ standard solution (mM) was used to express the T-AOC, which was determined from a standard curve to measure the antioxidant value.

### 4.7. Cell Culture and Viability Assay

HT22 cells were grown in DMEM supplemented with 10% fetal bovine serum, 100 μg/mL penicillin, and 100 μg/mL streptomycin. The cells were incubated at 37 °C in a 5% CO_2_ incubator. To ensure the absence of cytotoxicity induced by DVTP in HT22 cells, a density of 5 × 10^3^ cells per well was seeded in 96-well plates. Subsequently, DVTP was added to the culture medium at concentrations of 20, 40, 80, 160, or 320 μg/mL (excluding the normal control group), and the cells were incubated for 24 h. To assess the degree of damage caused by H_2_O_2_, various concentrations of H_2_O_2_ (100, 200, 300, 400, and 500 μM) were added to the culture media and incubated for 24 h. The control group was incubated without the addition of H_2_O_2_ for comparison. The cells were pretreated with various concentrations of DVTP in the medium for 4 h to investigate its ability to shield against damage caused by H_2_O_2_. Following pretreatment, 300 μM H_2_O_2_ was added for a duration of 24 h, excluding the control group. Cell viability was assessed via a CCK-8 test [71], where a CCK-8 solution (10%) was added to each well and incubated at 37.0 °C for 2 h before the absorbance at 450 nm was recorded via a microplate reader.

### 4.8. Statistical Analysis

Statistical analysis of the RSM data was performed via Design Expert 8.6. Other experimental data were statistically analyzed via GraphPad Prism 9.5.1. All the data are presented as means ± standard deviations, while the differences between groups were analyzed via one-way analysis of variance (ANOVA). Statistical significance was determined by considering *p* < 0.05 or *p* < 0.01.

## 5. Conclusions

In this study, the response surface model was used to optimize deproteinization, and the experimental results provided were close to the predicted scores, indicating that the prediction effect was good. The model designed in this study can be used to optimize the process of SuoSuo grape polysaccharide deproteinization. The structural characterization of DVTP revealed that DVTP is an acidic heteropolysaccharide rich in Gal-UA, and its antioxidant capacity is greater than that of VTP, indicating that proper deproteinization can effectively retain or even improve the biological activity of polysaccharides. CCK-8 assays also revealed that DVTP had a strong protective effect on HT22 cell injury induced by H_2_O_2_. The results showed that DVTP had good antioxidant properties and was suitable for use as a natural antioxidant. Unfortunately, molecular weight measurements indicate that DVTP is not a homogeneous polysaccharide, so further isolation and purification are needed to maximize the bioactivity of the SuoSuo grape polysaccharide. In addition, the exact antioxidant mechanism of DVTP is unknown, and further studies are needed to elucidate this phenomenon.

## Figures and Tables

**Table 1:** Box–Behnken center combination experimental design and results.

Run	A	B	C	D	Protein Removal Rate (%)	Polysaccharide Retention Rate (%)	Comprehensive Score
1	0	1	0	1	38.57 ± 0.64	71.67 ± 0.84	87.70
2	1	−1	0	0	45.97 ± 0.64	65.67 ± 2.34	91.46
3	−1	1	0	0	32.80 ± 0.35	71.08 ± 2.03	81.28
4	0	−1	0	1	47.83 ± 0.45	66.25 ± 3.41	93.79
5	−1	0	−1	0	32.14 ± 0.50	73.16 ± 6.45	81.96
6	0	0	0	0	42.44 ± 0.38	67.67 ± 0.68	89.10
7	0	0	0	0	41.95 ± 0.25	72.74 ± 1.58	91.94
8	−1	0	1	0	40.30 ± 0.65	67.75 ± 0.91	86.92
9	0	0	−1	−1	29.71 ± 0.55	73.21 ± 4.13	79.45
10	0	0	1	1	45.41 ± 0.59	67.53 ± 2.59	92.11
11	1	1	0	0	37.47 ± 2.17	69.09 ± 1.47	84.84
12	1	0	0	−1	39.14 ± 1.77	68.36 ± 0.80	86.11
13	−1	0	0	1	39.56 ± 0.31	70.44 ± 6.88	87.92
14	0	1	−1	0	31.97 ± 0.67	75.64 ± 1.05	83.42
15	0	0	−1	1	38.69 ± 0.43	68.01 ± 2.81	85.40
16	0	1	0	−1	32.69 ± 0.71	70.69 ± 2.79	80.90
17	0	−1	1	0	43.97 ± 0.33	69.85 ± 1.40	92.13
18	−1	0	0	−1	33.09 ± 0.37	66.77 ± 0.87	78.73
19	0	−1	0	−1	43.48 ± 1.77	66.79 ± 3.52	89.60
20	−1	−1	0	0	37.11 ± 0.05	68.44 ± 2.95	84.04
21	0	1	1	0	37.51 ± 0.61	70.58 ± 3.08	85.87
22	1	0	1	0	46.69 ± 0.81	63.60 ± 4.60	90.85
23	0	−1	−1	0	43.92 ± 0.90	64.28 ± 1.85	88.41
24	0	0	1	−1	39.50 ± 0.57	68.95 ± 2.53	86.87
25	0	0	0	0	41.97 ± 0.64	67.97 ± 2.05	88.81
26	1	0	−1	0	38.34 ± 1.51	67.03 ± 2.38	84.39
27	1	0	0	1	44.96 ± 1.09	66.71 ± 0.91	91.10

Note: chloroform/n-butanol ratio (*v*/*v*) (A), polysaccharide/Sevage reagent ratio (*v*/*v*) (B), number of deproteinizations (C), and shaking time for deproteinization (D).

**Table 2:** One-way analysis of variance for the comprehensive scores of the response surface quadratic model of *Vitis vinifera* L. SuoSuo total polysaccharide deproteinization.

Origin of Variance	Sum of Squares	Degrees of Freedom	Mean Square	*F*-Value	*p*-Value
Model	419.58	14	29.97	11.75	<0.0001
A	64.90	1	64.90	25.44	0.0003
B	104.56	1	104.56	40.99	<0.0001
C	83.90	1	83.90	32.89	<0.0001
D	110.21	1	110.21	43.21	<0.0001
AB	3.72	1	3.72	1.46	0.2505
AC	0.5691	1	0.5691	0.2231	0.6452
AD	4.41	1	4.41	1.73	0.2130
BC	0.4041	1	0.4041	0.1584	0.6976
BD	1.69	1	1.69	0.6636	0.4312
CD	0.1293	1	0.1293	0.0507	0.8257
A^2^	40.12	1	40.12	15.73	0.0019
B^2^	5.47	1	5.47	2.14	0.1689
C^2^	15.77	1	15.77	6.18	0.0286
D^2^	11.74	1	11.74	4.60	0.0531
Residual	30.61	12	2.55		
Lack of Fit	24.63	10	2.46	0.8231	0.6630
Pure Error	5.98	2	2.99		
Cor Total	450.19	26			

Note: chloroform/n-butanol ratio (*v*/*v*) (A), polysaccharide/Sevage reagent ratio (*v*/*v*) (B), number of deproteinizations (C), and shaking time for deproteinization (D).

**Table 3:** BBD factors and levels of deproteinization determined via the Sevage method.

Levels	Factors			
	A	B	C	D
−1	3:1	2:1	3	10
0	4:1	3:1	4	20
1	5:1	4:1	5	30

Note: chloroform/n-butanol ratio (*v*/*v*) (A), polysaccharide/Sevage reagent ratio (*v*/*v*) (B), number of deproteinizations (C), and shaking time for deproteinization (D).

## Data Availability

Dataset available on request from the authors.
